# A pan-cancer study of class-3 semaphorins as therapeutic targets in cancer

**DOI:** 10.1186/s12920-020-0682-5

**Published:** 2020-04-03

**Authors:** Xiaoli Zhang, Brett Klamer, Jin Li, Soledad Fernandez, Lang Li

**Affiliations:** Department of Biomedical Informatics, College of Medicine, The Ohio State University, 320B Lincoln Tower, 1800 Cannon Dr., Columbus, OH 43210 USA

**Keywords:** Class-3 semaphorins, Gene expression, Survival, Tumour suppressor or promoter, Immune subtype, Tumour microenvironment, Drug sensitivity

## Abstract

**Background:**

Initially characterized as axon guidance factors, semaphorins also have been implicated to have critical roles in multiple physiological and developmental functions, including the regulation of immune responses, angiogenesis, organ formation, and the etiology of multiple forms of cancer. Moreover, their contribution in immunity and the regulation of tumour microenvironment is becoming increasingly recognized. Here, we provide a comprehensive analysis of class-3 semaphorins, the only secreted family of genes among veterbrate semaphorins, in terms of their expression profiles and their association with patient survival. We also relate their role with immune subtypes, tumour microenvironment, and drug sensitivity using a pan-cancer study.

**Results:**

Expression profiles of class-3 semaphorins (SEMA3s) and their association with patient survival and tumour microenvironment were studied in 31 cancer types using the TCGA pan-cancer data. The expression of SEMA3 family varies in different cancer types with striking inter- and intra- cancer heterogeneity. In general, our results show that SEMA3A, SEMA3C, and SEMA3F are primarily upregulated in cancer cells, while the rest of SEMA3s are mainly down-regulated in the tested tumours. The expression of SEMA3 family members was frequently associated with patient overall survival. However, the direction of the association varied with regards to the particular SEMA3 isoform queried and the specific cancer type tested. More specifically, SEMA3A and SEMA3E primarily associate with a poor prognosis of survival, while SEMA3G typically associates with survival advantage. The rest of SEMA3s show either survival advantage or disadvantage dependent on cancer type. In addition, all SEMA3 genes show significant association with immune infiltrate subtypes, and they also correlate with level of stromal cell infiltration and tumour cell stemness with various degrees. Finally, our study revealed that SEMA3 genes, especially SEMA3C and SEMA3F may contribute to drug induced cancer cell resistance.

**Conclusions:**

Our systematic analysis of class-3 semaphorin gene expression and their association with immune infiltrates, tumour microenvironment and cancer patient outcomes highlights the need to study each SEMA3 member as a separate entity within each specific cancer type. Also our study validated the identification of class-3 semaphorin signals as promising therapeutic targets in cancer although further laboratory validation still needed.

## Background

Semaphorins constitute a large family of secreted or membrane-bound signaling proteins. In mammals, ~ 25 members have been identified, and they fall into eight classes based on their structural similarity [1, 2]. All members have a ~ 500 amino acids long conservative N-terminal SEMA domain signature, which contains sites for semaphorin dimerization and receptor binding. Although initially identified as axon guidance molecules, in recent years semaphorins have been recognized to have crucial roles in the regulation of diverse biological and pathophysiological processes, including angiogenesis, cancer tumourigenesis, inflammation, apoptosis, and immune response [3, 4]. These multifaceted functions are attributed to activation of downstream signaling cascades mainly mediated by cell surface receptors plexins and neuropilins [5].

Class-3 semaphorins (SEMA3) are the only group of secreted proteins among the vertebrate semaphorins, and they are further sub-grouped into seven members (SEMA3A to SEMA3G). Similar to other semaphorin members, this family of genes has been reported to exhibit pivotal roles in a broad spectrum of biological processes, and recently their function in the regulation of immune system, angiogenesis, local cancer spread and metastases are becoming increasingly prominent [6–12]. Recent studies have shown that SEMA3s are secreted from tumour cells as well as from macrophages and fibroblasts, thereby influencing cancer cells and their microenvironments through direct or indirect mechanism to regulate cell-cell communications in the tumour micro-environment or vasculature. SEMA3 family members have been reported to have both pro- and anti-tumour functions, which are cell type and context dependent [9, 12]. For instance, SEMA3A induces invasion and correlates with poor survival in both pancreatic and colon cancers, but inhibits tumour invasion and metastasis in patients with prostate, breast, and lung cancer [9, 13]. SEMA3B was found to play a pivotal tumour suppressor role in multiple cancers such as breast, gastric, and lung cancers [14–17]. SEMA3D is found to inhibit tumour growth in colorectal and breast cancers, but it correlates with metastatic disease and worse survival in patients with pancreatic cancer [15, 18, 19]. SEMA3E shows tumour promoter role in multiple cancer types such as gastric, breast, and pancreatic cancers [11, 12, 20–22]. In another study, both SEMA3D and SEMA3E was found to inhibit glioblastoma cell growth [23]. SEMA3C is frequently associated with invasion and metastasis, while SEMA3F was mainly reported to function as a tumour suppressor [7, 9–13, 24]. SEMA3G was reported to possess anti-migration and anti-invasion role in glioma [25]. However, there has been so far no systemic study of this family of genes in different human cancers, and each of the genes was only studied in a few types of cancers and most of those studies were performed using cell lines and/or animal models.

In the present study, we investigated the expression pattern of this gene family and their association with patient overall survival in primary tumours of 30 cancer types and one blood cancer AML using the TCGA pan-cancer data, and related their expression with tumour microenvironment and pharmacological activity. SEMA3F is the only family member whose expression is consistently increased in primary tumour samples as compared to the associated adjacent normal. All other SEMA3 family members show inconsistent up- or down-regulation in different cancer tumours, where SEMA3B, SEMA3D, SEMA3E, and SEMA3G are primarily down-regulated, and SEMA3A and SEMA3C are mainly up-regulated in the tested cancer types. The direction of association of gene expression with overall survival varies depending on the isoform queried and cancer type tested. Moreover, we found that SEMA3 family is associated with immune subtypes and tumour microenvironment while the degree of association also varies for different family members and cancer types. More interestingly, our results show that SEMA3A, SEMA3C, and SEMA3F are more closely clustered and associated with more aggressive immune subtypes and tumour stem-cell-like features, indicating a role in immune response and drug resistance, which may explain the results that both SEMA3C and SEMA3F are likely involved in cancer cell resistance to a number of chemotherapy drugs. Our study highlights the essential difference of the SEMA3s that exist between different tumour types and subtypes within each tumour type and the need to study each SEMAs as a separate entity.

## Results

### SEMA3 gene expression in pan-cancer

**Fig. 1:**
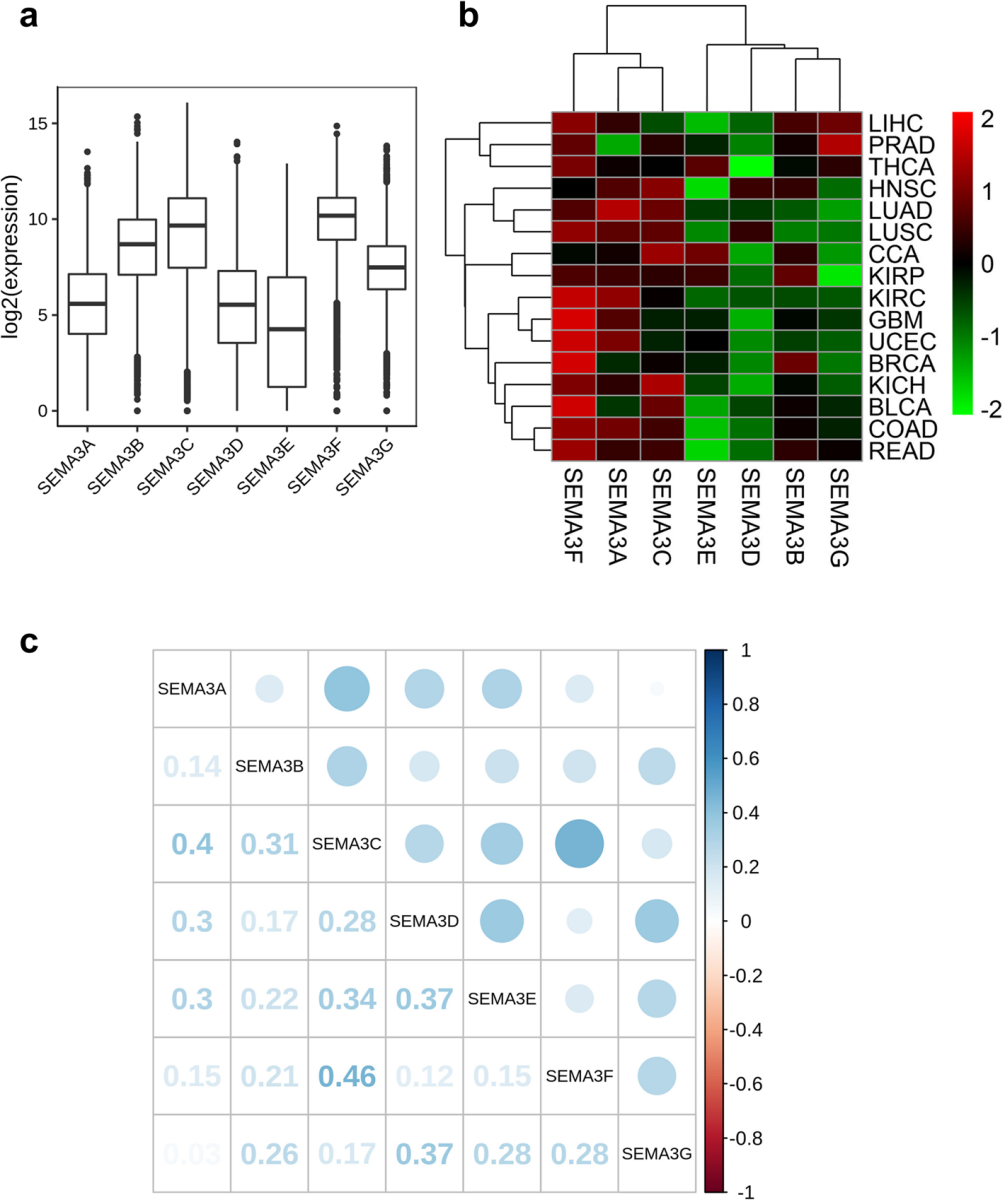
Expression levels of SEMA3 genes in cancerous and adjacent normal tissues. **a** Boxplot to show the distribution of SEMA3 gene expression across all 31 cancer types. **b** Heatmap to show the difference of SEMA3 gene expression comparing primary tumor to adjacent normal tisseus based on log2(fold change) for 16 cancer types that have more than 5 adjacent normal samples. **c** Correlation plot based on Spearman Correlation test results to show the correlation of gene expression among the 7 SEMA3 family members across all 31 cancer types

To understand the intrinsic expression pattern of SEMA3 genes, we examined the expression levels of the SEMA3 family members in all 31 cancer types available in TCGA pan-cancer data (Summery of TCGA data are in Additional file 3: Table S1 and Additional file 4: Figure S1). A striking intra- and inter-tumour heterogeneity regarding the expression levels of the corresponding genes were observed for all 7 SEMA3 members (Fig. 1a and Additional file 4: Figure S2). In terms of gene expression of a specific SEMA3 family member, there was striking heterogeneity of each gene expression across different tumour types with some tumour types expressing very high level of a specific gene, while others expressed negligible levels of that gene (Additional file 4: Figure S2). For example, expression levels of SEMA3E showed the largest inter-tumour heterogeneity with some tumours having very low levels of SEMA3E (ACC, DLBC, HNSC, KIRC, KIRP, AML, and PCPG), while others were characterized with high levels of SEMA3E expression (BRCA, CCA, and GBM) (Fig. 1a and Additional file 4: Figure S2). The other SEMA3 family members also showed various degrees of great heterogeneity. SEMA3A, SEMA3D, and SEMA3E showed relatively lower level of expression averaged across all cancer types compared to the rest of the SEMA3 family members, .i.e., SEMA3B, SEMA3C, SEMA3F and SEMA3G, where SEMA3F had the highest expression (Fig. 1a). These findings demonstrated the existence of intrinsic differences in the expression of semaphorin 3 gene family between different tumour types and between different SEMA3 family members within each tumour type, indicating a need for the study of each individual gene member as an entity.

One defining feature of genes that function during tumourigenesis is their dysregulated expression in cancer tumours, and accumulating evidence has shown that expressions of SEMA3 members are altered in multiple cancer types. However, much of the evidence has been based on work conducted on cell lines or animal models. In this study we investigated the expression levels of all 7 genes in primary patient tumours of 16 cancer types that have at least 5 paired adjacent normal samples (Fig. 1b and Additional file 4: Figure S2). All SEMA3s showed significant differential expression in different cancer types, however, the direction of the altered expression varies for each gene and for each cancer type (Fig. 1b). Whereas SEMA3A, SEMA3C, and SEMA3F were mainly up-regulated in the tested tumours, the rest of the members SEMA3B, SEMA3D, SEMA3E and SEMA3G were primarily down-regulated with a few exceptions. In addition, while the expression levels of various SEMA3 family members were positively correlated with each other averaged across cancer types based on Spearman Correlation tests, we found that the pairs SEMA3A and SEMA3C (*r* = 0.40, *P* < .0001), SEMA3C and SEMA3F (*r* = 0.46, *P* < .0001) had the highest correlation among all the pairwise correlations of the 7 genes, suggesting they may share some common features or functions (Fig. 1c).

### Association of SEMA3 gene expression with patient overall survival

**Fig. 2:**
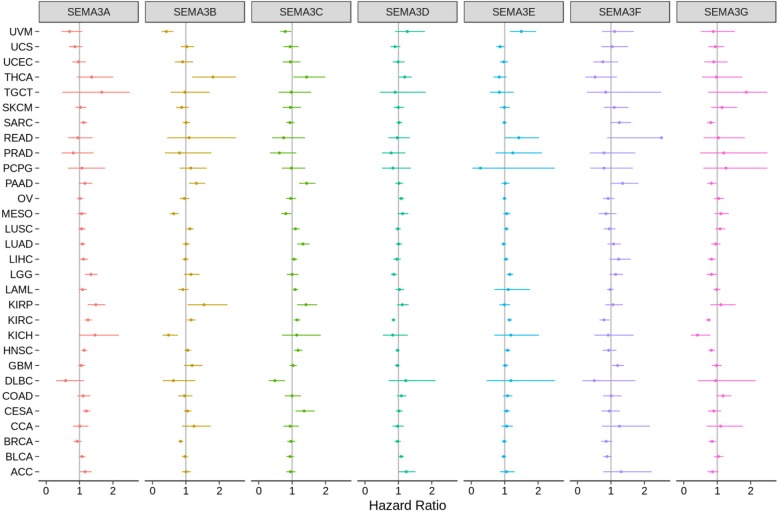
Association of SEMA3 gene expression with patient overall survival for different cancer types. The forest plots with the hazard ratios and 95% confidence intervals for overall survival for different cancer types to show survival advantage and disadvantage with increased gene expression of SEMA3 family. Univariate Cox proportional hazard regression models were used for the association tests

To correlate and eventually predict which semaphorin 3 family member promotes or inhibits tumourigenesis in which cancer type, the primary tumours from 29 cancer types and the blood samples from AML were used to investigate the association between SEMA3 gene expression and patient overall survival. This was analyzed using univariate Cox proportional hazard regression models and we claimed significant association with *P*-value< 0.05 without adjustment for multiple comparisons to be consistent with the display of forest plots in Fig. 2 (Test results are in Additional file 2). We found that the altered expression of SEMA3s were generally associated with patient overall survival, however the direction of the association varied depending on the member queried and the cancer type tested as shown in Fig. 2. More specifically, increased expression of SEMA3A and SEMA3E was mainly associated with increased survival disadvantage, where SEMA3A predicted poor prognosis of patients with SARC, LGG, LUAD, KIRP, KIRC, HNSC, CESA, and SEMA3E predicted poor prognosis for UVM, HNSC, KIRC, LGG, and READ. In contrast, increased expression of SEMA3G was primary associated with survival advantage and predicted better survival for patients with SARC, PAAD, LIHC, KIRC, KICH, HNSC, LGG and BRCA. The rest of SEMA3s were associated with either survival advantage or disadvantage depending on the cancer types. In more detail, SEMA3B predicted poor prognosis for KIRC, KIRP, LGG, LUSC, PAAD, and THCA, but predicted survival advantage for BRCA, KICH, MESO, and UVM. SEMA3C was associated with poor prognosis of patients with THCA, PAAD, LUAD, KIRP, KIRC, HNSC, CESA, and AML, but favored survival for patients with UVM, MESO, and DLBC. SEMA3D predicted poor prognosis for ACC, BLCA, OV, and THCA, but was associated with survival advantage for patients with LGG and KIRC. While increased SEMA3F expression only predicted poor prognosis for GBM and survival advantage for KIRC (Fig. 2 and Additional file 2). Worth noting is that among all the tested cancer types, all SEMA3 family members were significantly associated with overall survival of patients with kidney clear cell carcinoma (KIRC) (*P* < .02) (Fig. 2 and Additional file 4: Figure S3). However, the direction of the association was gene specific. Increased expression of SEMA3A, SEMA3B, SEMA3C and SEMA3E were associated with increased risk of survival, in contrast, increased expression of the rest of the SEMA3 family members was associated with reduced risk. Considering the genes are from the same family and may have redundant function, a multivariate cox proportional hazard regression model was used to investigate whether the significant association still exists when all members are considered in the same model. Results showed that SEMA3A, SEMA3D, SEMA3E, and SEMA3G were still significantly associated with patient survival (Additional file 3: Table S3).

### SEMA3 genes are associated with immune response and tumour microenvironment in cancer

**Fig. 3:**
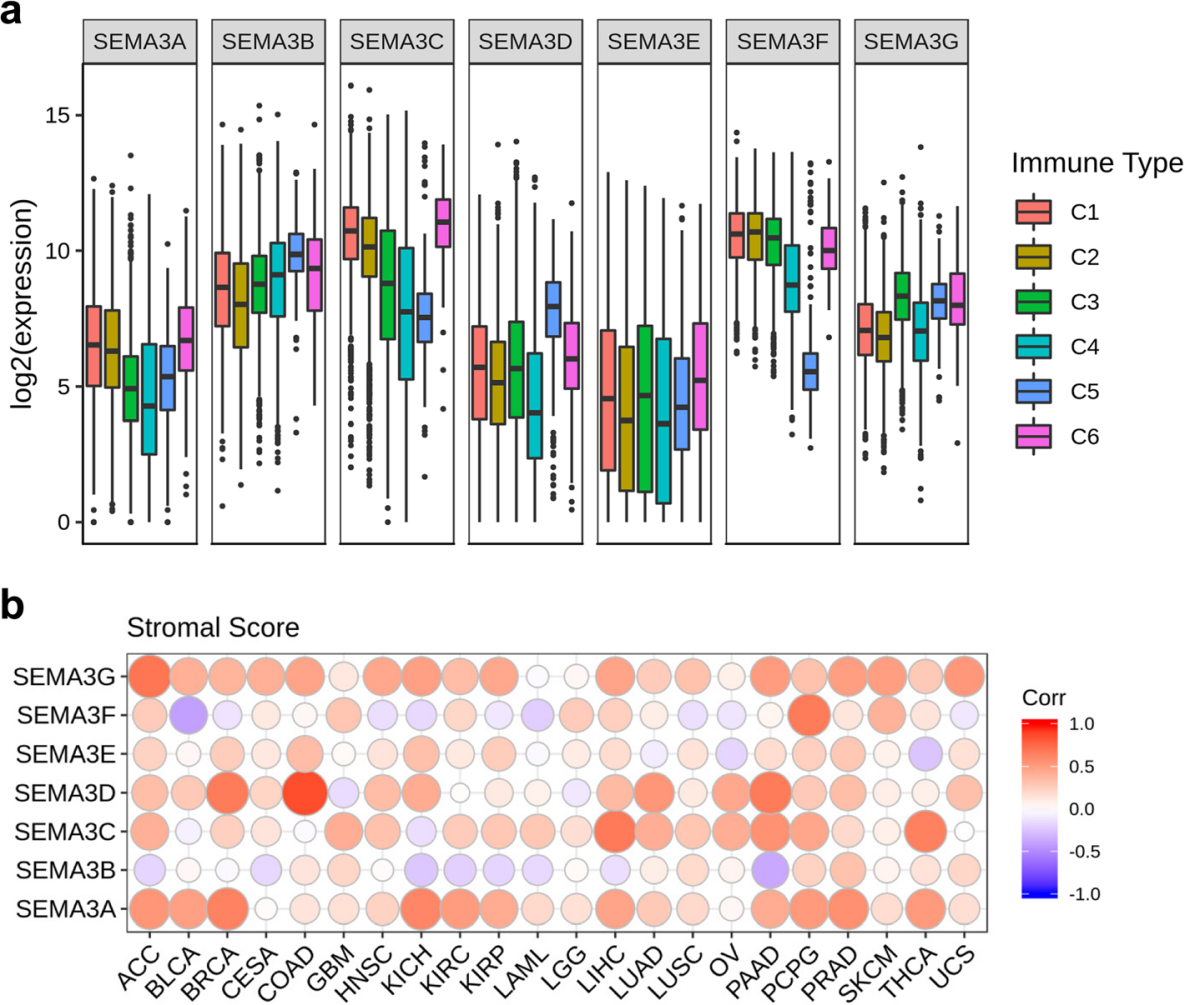
Association of SEMA3 gene expression with tumour microenvironment factors. **a** Association of SEMA gene expression with immune infiltrate subtypes across all the cancer types (*P* < .0001) tested with ANOVA. **b** Correlation matrix plots to show the association between SEMA3 gene expression and stromal scores of 22 different cancer types based on ESTIMATE algorithm. Spearman correlation was used for testing. The size of the dots stands for the absolute value of the correlation coeffcients. The bigger the size is, the higher the correlation is (higher absolute correlation coefficient). This also applies to Fig. 4a and b, as well as Additional file 4: Figure S4B and C

Semaphorins have been found to be crucially involved in various phases of immune responses, including regulating both immune cell interactions and immune cell trafficking during physiological and pathological immune responses, and those semaphorins are called “immuno” semaphorins [26]. SEMA3A and SEMA3E are among the so-called “immuno” semaphorins. In cancer context, SEMA3A is reported to play pleiotropic activities controlling tumour cells, tumour vessels and infiltrating inflammatory cells [24]. SEMA3E is found to regulate immune cell trafficking during the development of thymocytes, and it is also found to regulate NK-cell migration and NK–DC interactions [27, 28], but its role in the regulation of immune response in tumour microenvironment is not known. In order to understand how each of the SEMA3 family members is associated with immune components, we tested the correlation between SEMA3s and immune infiltrates in cancer tumours. Six types of immune infiltrates were identified in human tumours that correspond from tumour promoting to tumour suppressive, respectively [29]. They are C1 (wound healing), C2 (INF-r dominant), C3 (inflammatory), C4 (lymphocyte depleted), C5 (immunologically quiet), and C6 (TGFβ dominant). We analyzed immune infiltrates in the pan-can TCGA data and correlated them with the level of expression of SEMA3 family members (Fig. 3a). The overall survival across all cancer types showed that patients characterized into C3 and C5 immune subtypes had significantly better survival than patients characterized into other immune subtypes (*P* < 0.0001), where type C4 and C6 patients had least favorable survival (Additional file 4: Figure S4A) [29]. Correlations between higher levels of SEMA3A, SEMA3C, and SEMA3F and type 1, 2, and 6 infiltrates (C1, C2 and C6), indicated a tumour promoter role of these gene members, as patients belonging to those categories had worse survival characterized with higher proliferation rate and enriched with TGFβ (Fig. 3 and Additional file 4: Figure S4A). Similarly, SEMA3E had higher expression in C1 and C6 subtypes which associated with poor prognosis, indicating a tumour promoter role as well. In contrast, higher expression of SEMA3B and SEMA3D was correlated with C5 over other infiltrate types, and SEMA3G had significantly higher expression in components C3 and C5 than in C1, C2, and C4, suggesting an association of higher gene expression with favorable immune composition, which indicates that these genes may mainly play a tumour suppressor role.

As class-3 semaphorins can be secreted from tumour cells as well as macrophages and fibroblasts in the tumour microenvironment to regulate tumour growth and metastasis, we investigated the association between the expression levels of SEMA3s with the presence of infiltrating stromal cells in tumours indicated by stromal scores using the algorithm, ESTIMATE (Fig. 3b) [30, 31]. Not surprisingly, we also observed a wide range in the degree of association between SEMA3 family members and stromal score for different cancer types. SEMA3G showed the highest correlation with stromal score across cancer types (*r* = 0.38, *P* < .0001), followed by SEMA3C (*r* = 0.31, *P* < .0001), SEMA3A (*r* = 0.28, *P* < .0001), and SEMA3D (*r* = 0.22, *P* < .0001). The highest correlation between various SEMA3 family members and stromal score occurred in the following cancer types: ACC, BRCA, COAD, LIHC, KICH, PAAD, PCPG, PRAD, HNSC, and THCA. More specifically, we found significant positive correlation between SEMA3D and stromal score in COAD and PAAD, SEMA3A in KICH, SEMA3C in LIHC and PAAD, and SEMA3G in ACC (*P* < .0001). SEMA3F and SEMA3B showed significantly negative correlation with stromal score in BLCA and PAAD respectively (*P* < .0001). In addition, we also tested the correlation of SEMA3s with immune and estimate scores which measure the level of immune cell infiltrates and tumour purity in the tumours using ESTIMATE program [30, 31], and observed similar results to the stromal score tests (Additional file 4: Figures S4B and C).

### SEMA3 genes are associated with tumour stemness and cancer cell sensitivity to chemotherapy

**Fig. 4:**
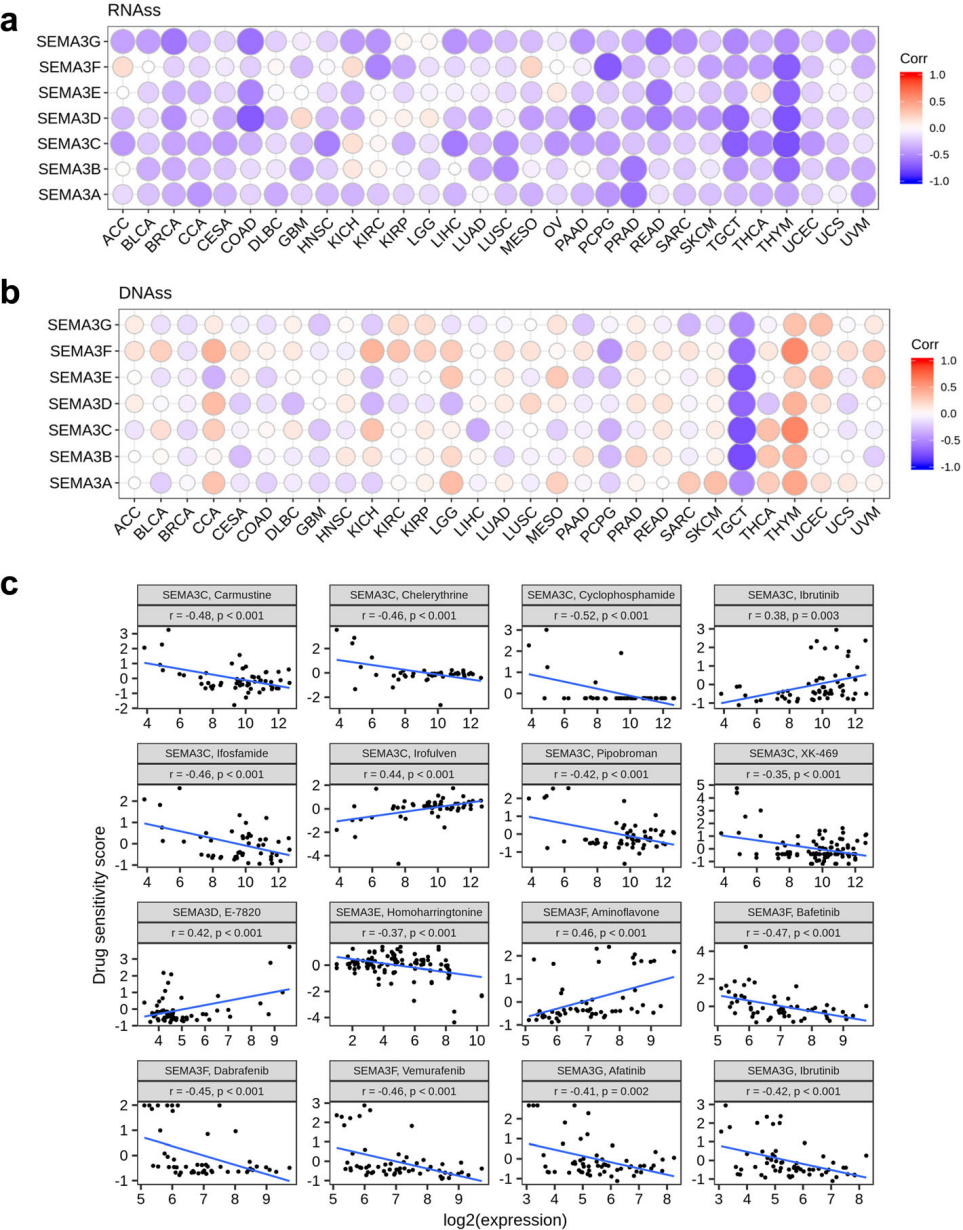
Association of SEMA3 gene expression with tumour stemness and drug sensitivity. **a** and **b** Correlation matrix between SEMA3 gene expression and cancer stemness scores RNAss (**a**) and DNAss (**b**) respectively based on Spearman correlation tests. **c** Scatter plots to show the association between SEMA3 gene expression and drug sensitivity (Z-score from CellMiner interface) tested with Pearson Correlation using NCI-60 cell line data

During cancer progression, tumour cells can gradually lose a differentiated phenotype and acquire progenitor and stem-cell-like features. SEMA3 members have been implicated having increased expression in tumour initiating stem cells and playing a role in tumour resistance. For instance, increased expression of SEMA3A reduces invasion and metastasis induced by the resistance to sunitinib in both cervical and pancreatic cancer [32]. SEMA3C is an indicator of poor prognosis in multiple cancer types and correlates with drug resistance in glioma, glioblastoma, and prostate cancer, possibly due to its high expression in cancer stem cells [33]. Tumour stemness can be measured with RNA stemness score based on mRNA expression (RNAss) and DNA stemness based on DNA methylation pattern (DNAss) [34]. The correlation between SEMA3 genes with tumour stemness measured by RNAss and DNAss was explored. SEMA3 family members showed various levels of association with RNAss and DNAss in different cancer types (Fig. 4a and b). Very interestingly, we found that SEMA3B, SEMA3D, SEMA3E, and SEMA3G genes were negatively associated with RNAss and DNAss (*P* < .0001), where the strongest association was observed for SEMA3G with RNAss (*r* = − 0.49) across cancer types. The other SEMA3 family members (SEMA3A, SEMA3C and SEMA3F) were either associated with RNAss with a negligible correlation coefficient (p-vlaue was significant) or insignificantly associated with RNAss, but they were all positively correlated with DNAss (*P* < .0001). Notably, although all genes were strongly negatively correlated with both DNAss and RNAss for TGCT, genes showed positive correlation with DNAss and negative correlation with RNAss in THYM. These contradictory results suggest that RNAss and DNAss may identify distinct cancerous cell populations characterized by different features or degrees of stemness in different cancers [34].

As SEMA3 genes are frequently associated with stem-cell-like features, we next investigated the SEMA3 gene expression in NCI-60 cell lines and systematically tested the correlation between their expression levels in the 60 human cancer cell lines (NCI-60) with drug sensitivity of over 200 chemotherapy drugs (Results are in Additonal file 3). We observed that levels of SEMA3 expression showed great heterogeneity in different cell lines as in patient tumours (Additional file 4: Figures S5A and B), with SEMA3C having the highest expression and SEMA3D having the lowest expression across all cancer cell lines. Drug sensitivity was measured by Z-score, and the higher the score is, the cells are more sensitive to the drug treatment (Additional file 4: Figure S5C). Interestingly, we found that increased expression of SEMA3s, especially SEMA3C and SEMA3F, were related with increased drug resistance of different cell lines to a number of chemotherapy drugs (*r* > 0.4 and *P* < .0001) (Fig. 4c). For example, SEMA3C was associated with cell resistance to the treatment of Pipobroman (treatment for CML), Ifosfamide (treatment for recurrent testicular cancer and germ cell tumours, sarcomas, Non-Hodgkin’s lymphoma, Hodgkin’s disease, non-small cell and small cell lung, cancer, bladder cancer, head and neck cancer, and cervix cancer), and Carmustine (treatment of brain tumours, multiple myeloma, Hodgkin’s disease, and non-Hodgkin’s lymphomas); and SEMA3Fwas associated with cell resistance to Vemurafenib (treatment for late-stage melanoma), Dabrafenib (treatment for late-stage melanoma and metastatic non-small cell lung cancer *with BRAF* V600E or V600K mutations), and Bafetinib (for CML) (Fig. 4c). We also noticed that a few genes were associated with drug sensitivity of several drugs. Moreover we noticed that different genes can have opposite associations with the same drug. For example, SEMA3C was associated with increased sensitivity of cells to ibrutinib (treatment for MCL and CLL), while SEMA3G was associated with increased resistance of cells to the same drug.

### SEMA3 gene family in breast cancer

**Fig. 5:**
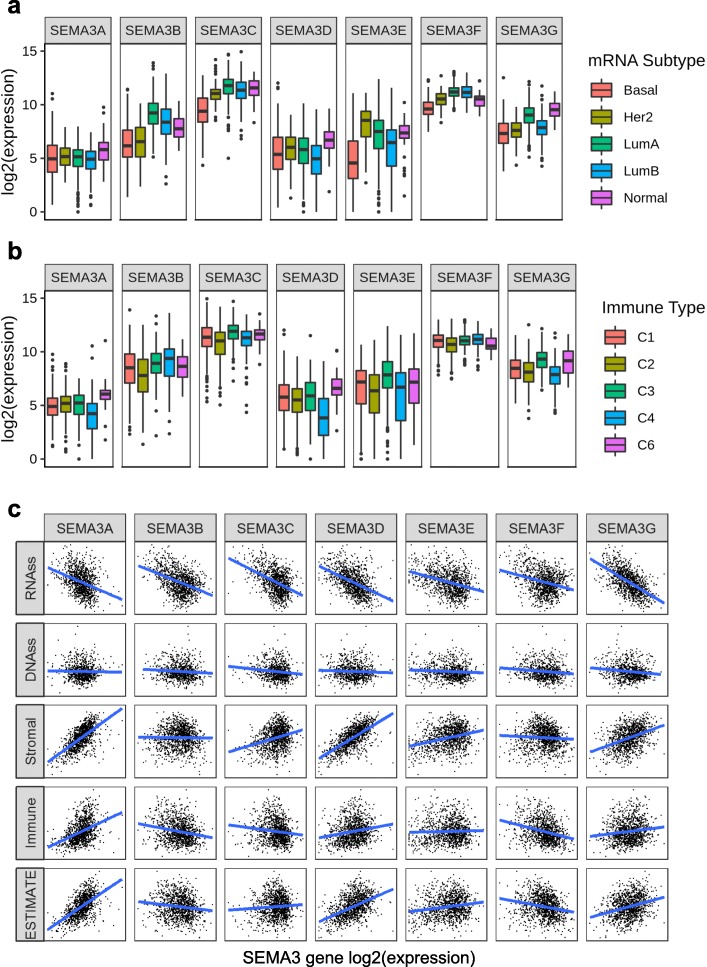
SEMA3 gene expression in breast cancer. **a** Association of SEMA3 gene expression with breast cancer molecular subtypes (*P* < .0001) tested with ANOVA. **b** Association of SEMA3 gene expression with immune infiltrate subtypes in breast cancer tested with ANOVA (*P* < .0001). **c** Correlation matrixes between SEMA3 gene expression and RNAss, DNAss, stromal score, immune score, and Estimate Score. Spearman correlation tests were used for testing

To our knowledge, with the exception of SEMA3G, all SEMA3 family members have been at least partially studied in breast cancer. Most members showed tumour inhibiting roles while only SEMA3E was found to promote tumour invasion and metastasis [13, 15, 20, 35–44]. However, as mentioned above, most of the studies were conducted in cell lines or animal models. Here we provide a thorough investigation of SEMA3 genes in one of the largest breast cancer patient cohort publicly available using TCGA breast cancer data. All SEMA3s except SEMA3B (*P* = 0.35) were significantly differentially expressed in breast cancer tumours compared to adjacent normal (*P* < .00001), where only SAEMA3F has significantly increased expression (Fig. 1 and Additional file 4: Figure S6A). Among the pairwise associations of the 7 genes, SEMA3A and SEMA3D (*r* = 0.59, *P* < .0001), SEMA3B and SEMA3F (*r* = 0.52, *P* < .0001) were more positively correlated (Additional file 4: Figure S6B). Considering the heterogeneity of tumours from the same origin, we further compared the expression of SEMA3s among the five breast cancer molecular subtypes (Fig. 5a). Interestingly, all the genes with decreased expression in breast cancer tumour showed significantly higher expression in normal like-tumours than in other more aggressive subtypes, i.e., Her2, LumA, LumB, and Basal type, except for SEMA3E, which also shows higher expression in Her2 type tumours, one of the most aggressive type of breast cancer subtypes. By contrast, SEMA3B had similar higher expression in normal-like, LumA, and LumB than in Her2 and Basal like tumours, and SEMA3F had significantly higher expression in Her2, LumA, LumB than in normal and basal type of breast cancer. As Her2 and basal like breast cancer are the more aggressive types of tumours, higher gene expression in these subtypes may associate with poor prognosis, such as in the case of SEMA3E and SEMA3F. Taken together, those results indicate the role of each specific SEMA3 family member in breast cancer may be breast cancer molecular subtype dependent based on the heterogeneity of individual tumours. This conclusion may also hold for other cancer types, such as BLCA, HNSC, KIRC and OV, as similar phenomenon that SEMA3 gene expressions vary in different molecular subtypes within the same tumor origin were also observed in these cancer types (Additional file 4: Figure S7).

A possible alternative mechanism governing alterations in SEMA3 expression in tumours is that SEMA3s are differentially expressed by various cell types in the tumour microenvironment. The association between SEMA3 gene expression and immune subtypes in breast cancer had a similar pattern compared to that observed using all 31 TCGA tumours across cancer types; and all 7 genes were significantly associated with immune infiltrate types (*P* < .0001), although no patient sample belonged to C5 immune subtype in breast cancer (Figs. 3 and 5a and b). As stromal cells are a big compartment in the tumour microenvironment especially in breast cancer, we further looked into the SEMA3 expression and stromal score correlation. We found that SEMA3A, 3C, 3D, 3E and 3G were all positively correlated with stromal scores (*P* < .0001) in breast cancer where SEMA3A and SEMA3D had the strongest correlation (*r* > 0.6) (Figs. 3 and 5c). In contrast, SEMA3B and SEMA3F had no significant correlation with stromal score, indicating SEMA3A, 3C, 3D, 3E and 3G may have been expressed by tissue stroma in breast cancer. SEMA3A and SEMA3D were also found to be correlated with immune score which measures the presence of infiltrating immune cells (*P* < .0001) and tumour purity (Estimate score) (*P* < .0001) (Additional file 4: Figures S4B and 5C).

All SEMA3 gene expressions favored better survival with increased expression, although only SEMA3B and SEMA3G showed significant association (Fig. 2). SEMA3 family members except for SEMA3F were all negatively associated with RNA stemness score (*r* = − 0.32 to − 0.65, and *P* < .0001), as well as a smaller degree of association with DNAss (*P* < 0.03) (Fig. 5c). These results that higher expression of SEMA3s correlates with reduced cancer cell stemness in breast cancer are consistent with the fact that increased expression of SEMA3s favored better survival, as lower tumor stemness generally correlates with better survival.

## Discussion

In the past decade, accumulating evidence has shown that class-3 semaphorins and their receptors play pivotal roles during tumour growth and metastatic spread, and they have been found to be involved in controlling tumour cell viability, apoptosis, proliferation, adhesion, migration, and invasion. More importantly, SEMA3s were found to either promote or inhibit tumour growth by influencing both the tumour compartment and its micro-environment, and they have been extensively studied as therapeutic targets in cancer.

Our study provides a first systemic pan-cancer analysis of the class-3 semaphorins. We found great heterogeneity of the levels of SEMA3 gene expression among different tumour types and within each tumour type. Among them, SEMA3E showed the greatest inter- tumour heterogeneity. In addition to the observation that expression of a specific isoform of SEMA3 gene vary greatly among tumours, we found that their expression varied even within each histological subtype/tissue of origin, such as in the cases of gene expression variation in different molecular subtypes of BRCA, HNSC, BLCA, OVand KIRC, as well as in different immune subtypes. Nevertheless, SEMA3A, SEMA3C, and SEMA3F primarily showed up-regulated expression, and the rest of the SEMA3s mainly showed down-regulated expression in the 16 tested cancer types. We further tested the association between the levels of expression of SEMA3s with patient overall survival in 30 cancer types, and found that the direction of association is also cancer type dependent, but in general SEMA3A and SEMA3E were mainly associated with poor prognosis, and SEMA3G was associated with better survival, while the rest of the SEMA3s had an antagonistic association with survival (both advantage and disadvantage). Interestingly, our study found that all SEMA3 family members significantly associated with immune infiltrate subtypes in tumour microenvironment, where SEMA3A, SEMA3C, SEMA3E, and SEMA3F correlated with more aggressive subtypes of immune infiltrates, i.e., C1, C2, and C6, indicating a correlation with poor prognosis. SEMA3s also correlated with level of stromal cell infiltrates and immune cell infiltrates with various degrees based on the ESTIMATE algorithm. Those findings are consistent with the results of past observations that SEMA3s can function as pro-inflammatory and immune modulators [3, 12, 13, 24], and they may be used as direct therapeutic targets or help predict the efficacy of immune checkpoint modulators in cancer patients. The association between SEMA3 gene expressions and tumor stemness score as well as with the drug sensitivity score indicated that SEMA3A, SEMA3C, and SEMA3F may mainly play tumor promotor roles during tumorigenesis as they are positively associated with tumor stemness and drug resistence scores. Furthermore, as mentioned above we observed that epxresssions of SEMA3 vary in different molecular subtypes within the same tumor origin and the tumor suppressor or promotor role could be subtype specific. For instance, although expressions of all SEMA3 genes are associated with a better prognosis based on the survival analyses in breast cancer, SEMA3E and SEMA3F was found to be associated with more aggressive HER2 and basal like molecular subtypes, indicating a tumor promoter role in those subtypes. However, all the findings need to be further validated in laboratory although the results show SEMA3s could be promising therapeutic targets based on the association studies.

Among the 7 SEMA3 gene members, expression levels of SEMA3A and SEMA3C, SEMA3C and SEMA3F showed the highest correlation across all 31 cancer types, suggesting they may share some common functions. Indeed, we found that they not only had increased expression in most of the 16 tested cancer types, but they also had similar correlation pattern with immune infiltration subtypes, where they were highly expressed in immune subtypes (i.e., C1, C2, and C6) with high proliferation rate and enriched with IFN-r infiltrates, indicating a tumour promoter role. Interestingly, although the rest of SEMA3 family members showed significant negative correlation with tumour stem-cell-like features measured by mRNA (RNAss) and DNA methylation (DNAss), expression of SEMA3A, SEMA3C, and SEMA3F were not significantly correlated with RNAss. Instead, they were positively correlated with stem-cell-like features measured with DNA methylation (DNAss), suggesting they likely play a role in tumour initiating cells and relate with tumour resistance to drug treatment. SEMA3A has been extensively studied and it shows both pro- and anti-tumour effects [12, 13, 24]. Similar to previous reports, we found that SEMA3A was either up- or down-regulated in different cancer types, but we only found SEMA3A associated with poor prognosis in a number of cancer types (SARC, LUAD, HNSC, LGG, KIRC, and KIRP), where the expression of SEMA3A in tumours were all up-regulated. Therefore, the role of SEMA3A as tumour suppressor needs to be reevaluated. Recent studies have shown that SEMA3C plays an oncogenic role and associated with poor prognosis and progression in multiple cancer types, including breast cancer [12, 13, 24]. Moreover, SEMA3C has been demonstrated to play a key role in maintaining stem-cell-like cell features in glioblastoma and prostate cancer [13, 33, 45, 46]. However, our study showed that altered expression of SEMA3C associated with poor prognosis in THCA, KIRC, KIRP, HNSC, PAAD, LUAD and CESA, but also associated with survival advantage in UVM, MESO, and DLBC which is all cell context dependent. Regarding the function of SEMA3C in breast cancer, our study showed SEMA3C was down-regulated in primary tumours with further reduced expression in the aggressive subtype Her2 and basal-like tumours, indicating a tumour suppressor role of SEMA3C in breast cancer. However, this finding contradicts previous reports where SEMA3C is expressed more highly in Her2 and triple negative breast cancer tumours [47] and associates with tumour growth and invasion. The discrepancy could be due to the fact that our study is based on SEMA3C expression at mRNA level while the previous study measured SEMA3C expression at protein level in tissue microarrays. Therefore, the role of SEMA3C either functioning as a tumour promoter or suppresser in breast cancer still needs to be validated in future studies. SEMA3F has been reported to function as a tumour suppressor inhibiting tumour growth and invasion in a number of cancer types, including lung, prostate, bladder, osteosarcoma, ovarian cancer, and melanoma cancer [3, 7, 12, 13, 24]. Interestingly although SEMA3F was up-regulated in most of the 16 tested cancer types except in HNSC, LUAD, and PRAD, the increased expression of SEMA3F was generally not associated with overall survival except with survival advantage in KIRC and poor prognosis in GBM, but the correlation studies between SEMA3F expression and immune components, as well as with tumor stemness indicate a tumor promotor role of SEMA3F in tumors. The contradictory results of SEMA3F with increased expression in cancer tumours while function as tumour suppressor under certain circumstances such as in KIRC needs to be further studied. In addition, the discrepancy comparing our results that SEMA3F had increased expression in tumors and possible tumor promoting role to previous reports could be due to the fact that the data were from different patient cohorts, as well as that many previous studies were based on cell line experiments. Therefore, the function of SEMA3F as a tumor promotor or tumor suppressor still needs to be further studied. The pheonomenon of increased gene expression in cancer tumours while function as tumour suppressor or of decreased gene expression while function as tumor promotor has also been observed for SEMA3E and other genes as well. In the case of SEMA3E, it had down-regulated expression in most of the tested cancer types, but it mainly associated with poor prognosis. Therefore, the predicted role of each gene as tumor suppressor or promoter in a specific cancer type needs to be validated using experiments.

In contrast to SEMA3A, SEMA3C, and SEMA3F, the rest of the SEMA3 family members showed prominent down-regulation and they all negatively correlated with cancer stem-cell-like features (RNAss and DNAss) across cancer types. Among them, SEMA3D, SEMA3E, and SEMA3G were more tightly correlated than with SEMA3B. Although our findings are similar to previous studies about SEMA3B and SEMA3D with both pro- and anti-tumoural activities, and SEMA3G mainly playing anti-tumoural roles, we observed that SEMA3E mainly associated with poor prognosis as opposed to previous reports that it can have a dual role and suppress tumour progression under certain circumstances [3, 7, 9, 12, 13, 24, 26]. In addition, we found that SEMA3B and SEMA3D had significantly higher expression in immune infiltrate C5 than in other immune subtypes, and SEMA3G had higher expression in C3 and C5, suggesting an association with favorable immune composition as patients characterized into these two immune subtypes having significantly better survival. In contrast, SEMA3E had higher expression in immune infiltrates C1 and C6 which associated with poor prognosis. This phenomenon may explain the above finding that SEMA3E likely promotes tumour growth in cancer. In addition, SEMA3D and SEMA3G showed relatively strong correlation with stromal score, suggesting they may be secreted by stromal cells or have role in stromal related activities.

Cancer stem-like-cells (CSC) can arise from different sources including from long-lived stem or progenitor cells or via dediffentiation from non-stem cancer cells that convert to CSCs through deregulation of related signaling pathways [34]. CSCs promote cancer progression due to the capacity for self-renewal and invasion, and it is the main cause for treatment induced drug resistance [48–50]. In the present study, we investigated the expression of SEMA3s with stem-cell-like features measured with RNAss and DNAss [34]. As mentioned above, we found that SEMA3B, SEMA3D, SEMA3E, and SEMA3G were all negatively associated with cancer-stem like features, but SEMA3A, SEMA3C, and SEMA3F were positively correlated with DNAss, which indicates that SEMA3s may associate with cancer cell sensitivity or resistance to chemotherapy treatment. By using NCI-60 cell line data, we found that increased expression of SEMA3s, especially SEMA3C and SEMA3F, was associated with increased drug resistance for a number of FDA approved chemotherapy drugs, such as Pipobroman, Ifostamine, Carmustine, Vemurafenib, Dabrafenib, Ibrutinib, and Bafetinib (*r* = <− 0.38 and *p* < .0001). Of course, various members of SEMA3s were also associated with increased drug sensitivity of a few drugs. For example, increased expression of SEMA3C associated with increased cancer cell sensitivity to Irofulven and Ibrutinib, and SEMA3F associated with Aminoflavone. These data suggest that SEMA3s may play a role in cancer cell sensitivity or resistance to drug treatment and can be used as therapeutic targets to overcome drug induced resistance or to aid drug sensitivity.

## Conclusion

We provide a comprehensive and systematic characterization of the profiles of all the seven class-3 semaphorin genes in this pan-cancer study and demonstrate the need for studying their role in a cancer type, immune subtype, and molecular-subtype dependent manner. In summary, our results indicate that SEMA3A, SEMA3C, SEMA3E, and SEMA3F are more often to promote tumorigenesis and associate with poor prognosis, while the other SEMA3s are more often to play a tumour suppressor role and generally associate with better prognosis. However, the putative tumour promoter or tumour suppressor role of SEMA3 is not consistent among the SEMA3 family members within a specific cancer type, and also the specific SEMA3 isoform behaves differently in different cancer types, and even in different cancer subtypes. Taken together, our work will greatly help in uncovering their role in tumorigenesis, specifically in immune response, tumour microenvironment and drug resistance, essential for developing personalized medicine for cancer treatment.

## Methods

### TCGA pan-cancer data

TCGA pan-cancer data, including RNA-Seq (RNA SeqV2 RSEM), clinical data, stemness scores based on mRNA (RNAss) and DNA-methylation (DNAss), and immune subtypes were downloaded from xena browser (https://xenabrowser.net/datapages/). The tumour samples in TCGA are surgical resection samples obtained from primary tumours that have received no prior neoadjuvant treatment. For inter-tumour/pan-tumour analyses, gene expression was normalized to TBP (TATA-box binding protein). The TCGA pan-cancer data include 31 cancer types, and they are ACC, BLCA, RCA, CCA, CESC, COAD, DLBC, GBM, HNSC, KICH, KIRC, KIRP, LAML, LGG, LIHC, LUAD, LUSC, MESO, OV, PAAD, PCPG, PRAD, READ, SARC, SKCM, TGCT, THCA, THYM, UCEC, UCS, UVM (Additional file 3: Table S1). In total 9445 samples were available for this study, and the number of samples available for each cancer type ranged from 45 for colangiocarcinoma to over 1000 for breast cancer (Additional file 4: Figure S1). Among them, 15 cancer types had none or less than 5 associated normal tissue samples, so only the rest of the 16 cancer types were used to investigate whether there was altered gene expression in tumours compared to adjacent normal with linear mixed effects models (Additional file 3: Table S1). In order to investigate the association between gene expression (as continuous variable) of each of the SEMA3 family members and patient overall survival, all patient tumour samples except THYM which had no patient survival information were used in a survival analysis. The summary of the overall survival event of different cancer types is listed in Additional file 3: Table S1 and Additional file 4: Figure S1.

### Tumour microenvironment analysis

The ESTIMATE immune score and stromal score were used to analyze the infiltration levels of immune cells and stromal cells in different tumours [31] . The estimate score from this program was used to describe tumour purity. This analysis was based on the interpretation of gene expression profiles retrieved from TCGA expression data (http: //bioinformatics.mdanderson.org/estimate/) [31]. Association between SEMA3 expression and those scores was tested with Spearman correlation. Six immune subtypes were defined to measure immune infiltrates in tumour environment [51]. Immune subtype obtained from TCGA pan cancer data was used to test the association between SEMA3 expression and immune infiltrate types in tumour microenvironment using ANOVA models. Tumour stemness features extracted from transcriptomic and epigenetic from TCGA tumour samples were used to measure stem-cell-like features of tumour cells [34]. The correlation of cancer stemness with SEMA3 expression was tested using Spearman correlation test.

### NCI-60 analysis

The NCI-60 database, which contains data on 60 different cancer cell lines from nine different types of tumours, was accessed using the CellMiner interface (https://discover.nci.nih.gov/cellminer/). SEMA3 mRNA expression levels and z scores for cell sensitivity data (GI50) were retrieved for 59 cell lines and were analyzed using Pearson correlation to investigate the relationship between gene expression and drug sensitivity. The drug response of 262 FDA approved or drugs on clinical trials were used in the correlation analysis.

### Statistical analyses

Comparison of gene expression between the normal and tumours were performed in 16 cancer types which had more than 5 associated adjacent normal samples using linear mixed effects models. Boxplots were used to show the gene expression across cancer types. Univariate or multivariate cox proportional hazard regression models or Log-rank tests were used to test the association between gene expression and patient overall survival. Spearman or Pearson correlation was used to test the correlation between gene expression and stemness scores, stromal score, immune score, estimate score, and drug sensitivity. Linear regressions were used to test the association between gene expression and patient clinical characteristics, immune components, breast cancer subtypes. All tests were performed using SAS9.4 (SAS Institute Inc., NC). Plots were created using R (R Core Team) with packages ggplot2, pheatmap, corrplot, or survminer where appropriate [52]. We used the number of false positives method to adjust for multiple comaprisons for all the tests, except for the survival study, to control the familywise error rate at α = 0.05 [53]. In more detail, we assumed 1 false positive among all the tests within each study to set the *p*-value cutoff for significance. For example, we used α = 1/(7 × 16) = 0.009 as cutoff for comparing SEMA3 gene expression in tumour to adjacent normal as there were 112 tests (7 genes × 16 cancer types). For the survival study, we used α = 0.05 as cutoff without addjsuting for multiple comparisons in order for the data interpretation to be consistent with the display of the results with forest plots. However, to claim significant assocaiton between gene expression and OS, the same method controlling for multiple comaprisons as for comparing gene expression beween tumor and normal was used, i.e., *p* < 1/(7 genes × 30 cancer types) = 0.005 was considered as significant.

## Supplementary information

**Additional file 1:** This is an MS Excel file containing the compiled TCGA data used for this study.

**Additional file 2:** This is an MS Excel file containing the SEMA3 gene expression and drug sensitivity score derived from NCI60 cell line study.

**Additional file 3: Table S1.** Summary of TCGA pan-cancer data including number of total samples, number of primary tumor and adjacent normal tissues, and number of overall survival event and censored patients. **Table S2:** Summary of the association between SEMA3 gene expressions with patient overall survival in different cancer types for the TCGA pan-cancer data. **Table S3.** Results of multivariate Cox Proportional hazard regression model to test the association between SEMA3 gene expression and overall survival of KIRC including the expression of all 7 SEMA3 genes.

**Additional file 4: Figure S1. (A)** Histogram to show the number of samples for primary tumor and adjacent normal tissues in each cancer type and the sample number for blood cancer AML. **(B)** Histogram to show number of death event, number of censored, and number of not available for the overall survival analysis for each cancer type. **Figure S2.** Expression levels of SEMA3 genes in cancerous and adjacent normal tissues for all 31 cancer types. Boxplots represent the distribution of the SEMA3 gene expression levels (log2[RSEM normalized values relative to TBP]) in primary tumour and normal tissues (if available) of different cancer types for each of the SEMA3 genes. The band inside the box is the median expression values for the gene. Comparisons between normal and tumour expression values were performed with linear mixed effects models. A *p*-value< 0.009 after controlling 1 false positive among all the tests was considered as significance (53). **Figure S3.** Kaplan-Meier survival curves to show the correlation between SEMA3 gene expression and overall survival of patients with kidney clear cell carcinoma (KIRC). Gene expression was dichotomized into “Low” and “High” based on the median expression of each specific gene in KIRC and overall survival was tested between patients with low and high gene expression using log-rank tests. Worthnoting is that in **Figure S3.** the *p*-values are different from that of the Cox-proportional hazard model where gene expression was used as a continuous variable and all 7 genes were significantly associated with overall survival of KIRC. **Figure S4.** (A). Kaplan-Meier survival curve to show the overall survival difference among the six immune subtypes across all cancer types. (B) and (C). Correlation matrix plots to show the association between SEMA3 gene expression and immune scores (B), and Estimate scores (C), of 22 different cancer types based on ESTIMATE algorithm. Spearman correlation was used for testing. The size of the dots stands for the absolute value of the correlation coeffcients. The bigger the size is, the higher the correlation is (higher absolute correlation coefficient). **Figure S5.** (A). Boxplots to show the distribution of SEMA3 gene expression across NCI-60 cell lines. (B). Heatmap to show SEMA3 gene expression within each individual cell lines using NCI-60 cell line data. (C). Boxplots to show the sensitivity score distribution of the FDA approved chemotherapy drugs that are significantly associated with SEMA3 gene expression. **Figure S6.** (A). Boxplot to show the distribution of SEMA3 gene expression comparing primary tumor to adjacent normal in breast cancer. (B). Correlation plot to show the correlation of gene expression among the 7 SEMA3 family members in breast cancer. **Figure S7.** Boxplots to show the association of SEMA3 gene expressions with molecular subtypes of BLCA, HNSC, KIRC, and OV tested with ANOVA (*P* < .0001).

## Data Availability

The compiled TCGA dataset and the NCI 60 cell line data supporting the conclusions of this article are included within the article (Additional files 1 and 2).

## Abbreviations

ANOVA: Analysis of variance
DNAss: Tumor stemness based on DNA methylation
r: Correlation coefficient
RNAss: Tumor cell stemness based on mRNA expression
SEMA3: Class-3 semaphorins
TCGA: The cancer genome atlas
TCGAID: For the cancer type abbreviations (TCGAID) please refer to Additional file 3: Table S1.
