# Supplementary material for: A pan-cancer study of class-3 semaphorins as therapeutic targets in cancer
Source: BMC Med Genomics. 2020 Apr 3;13(Suppl 5):45. doi: 10.1186/s12920-020-0682-5 (PMC7118829; doi:10.1186/s12920-020-0682-5)
Supplement: Supplementary file 3 — Additional file 3: Table S1. Summary of TCGA pan-cancer data including number of total samples, number of primary tumor and adjacent normal tissues, and number of overall survival event and censored patients. Table S2: Summary of the association between SEMA3 gene expressions with patient overall survival in different cancer types for the TCGA pan-cancer data. Table S3. Results of multivariate Cox Proportional hazard regression model to test the association between SEMA3 gene expression and overall survival of KIRC including the expression of all 7 SEMA3 genes. [file 12920_2020_682_MOESM3_ESM.pptx]

## Slide 1
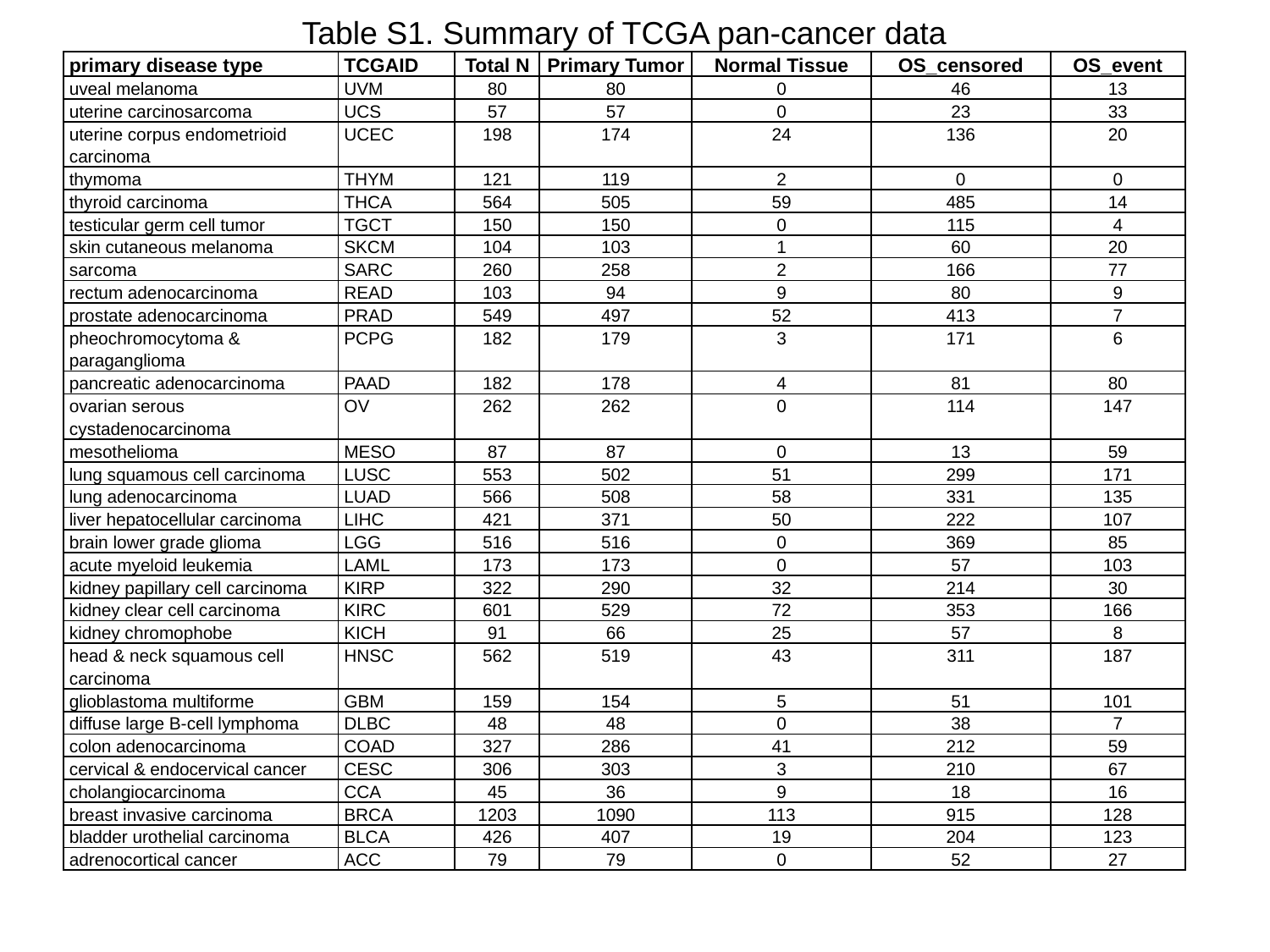

| Table S1. Summary of TCGA pan-cancer data | | | | | | |
| --- | --- | --- | --- | --- | --- | --- |
| primary disease type | TCGAID | Total N | Primary Tumor | Normal Tissue | OS\_censored | OS\_event |
| uveal melanoma | UVM | 80 | 80 | 0 | 46 | 13 |
| uterine carcinosarcoma | UCS | 57 | 57 | 0 | 23 | 33 |
| uterine corpus endometrioid carcinoma | UCEC | 198 | 174 | 24 | 136 | 20 |
| thymoma | THYM | 121 | 119 | 2 | 0 | 0 |
| thyroid carcinoma | THCA | 564 | 505 | 59 | 485 | 14 |
| testicular germ cell tumor | TGCT | 150 | 150 | 0 | 115 | 4 |
| skin cutaneous melanoma | SKCM | 104 | 103 | 1 | 60 | 20 |
| sarcoma | SARC | 260 | 258 | 2 | 166 | 77 |
| rectum adenocarcinoma | READ | 103 | 94 | 9 | 80 | 9 |
| prostate adenocarcinoma | PRAD | 549 | 497 | 52 | 413 | 7 |
| pheochromocytoma & paraganglioma | PCPG | 182 | 179 | 3 | 171 | 6 |
| pancreatic adenocarcinoma | PAAD | 182 | 178 | 4 | 81 | 80 |
| ovarian serous cystadenocarcinoma | OV | 262 | 262 | 0 | 114 | 147 |
| mesothelioma | MESO | 87 | 87 | 0 | 13 | 59 |
| lung squamous cell carcinoma | LUSC | 553 | 502 | 51 | 299 | 171 |
| lung adenocarcinoma | LUAD | 566 | 508 | 58 | 331 | 135 |
| liver hepatocellular carcinoma | LIHC | 421 | 371 | 50 | 222 | 107 |
| brain lower grade glioma | LGG | 516 | 516 | 0 | 369 | 85 |
| acute myeloid leukemia | LAML | 173 | 173 | 0 | 57 | 103 |
| kidney papillary cell carcinoma | KIRP | 322 | 290 | 32 | 214 | 30 |
| kidney clear cell carcinoma | KIRC | 601 | 529 | 72 | 353 | 166 |
| kidney chromophobe | KICH | 91 | 66 | 25 | 57 | 8 |
| head & neck squamous cell carcinoma | HNSC | 562 | 519 | 43 | 311 | 187 |
| glioblastoma multiforme | GBM | 159 | 154 | 5 | 51 | 101 |
| diffuse large B-cell lymphoma | DLBC | 48 | 48 | 0 | 38 | 7 |
| colon adenocarcinoma | COAD | 327 | 286 | 41 | 212 | 59 |
| cervical & endocervical cancer | CESC | 306 | 303 | 3 | 210 | 67 |
| cholangiocarcinoma | CCA | 45 | 36 | 9 | 18 | 16 |
| breast invasive carcinoma | BRCA | 1203 | 1090 | 113 | 915 | 128 |
| bladder urothelial carcinoma | BLCA | 426 | 407 | 19 | 204 | 123 |
| adrenocortical cancer | ACC | 79 | 79 | 0 | 52 | 27 |

## Slide 2
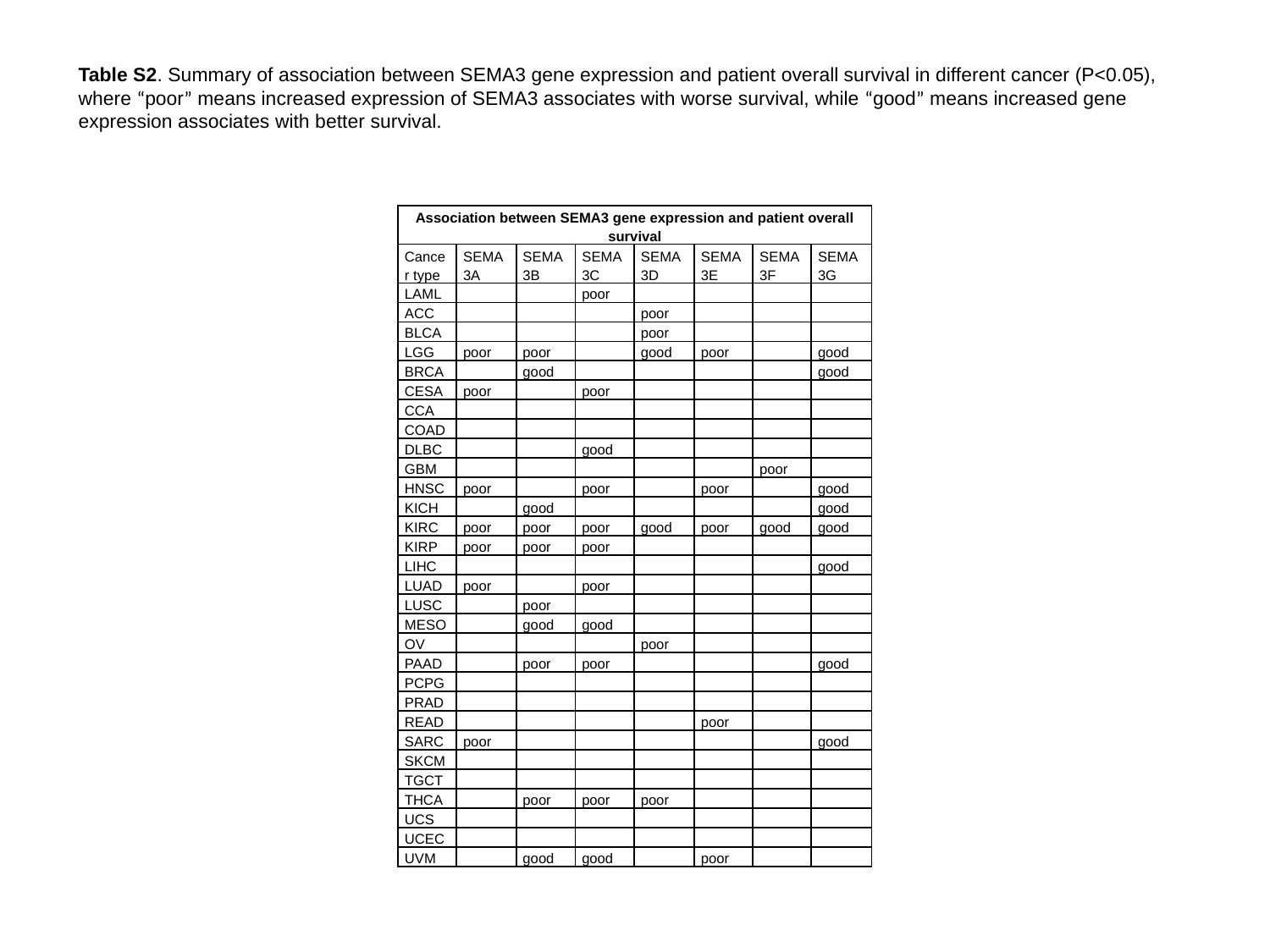

Table S2. Summary of association between SEMA3 gene expression and patient overall survival in different cancer (P<0.05),
where “poor” means increased expression of SEMA3 associates with worse survival, while “good” means increased gene
expression associates with better survival.
| Association between SEMA3 gene expression and patient overall survival | | | | | | | |
| --- | --- | --- | --- | --- | --- | --- | --- |
| Cancer type | SEMA3A | SEMA3B | SEMA3C | SEMA3D | SEMA3E | SEMA3F | SEMA3G |
| LAML | | | poor | | | | |
| ACC | | | | poor | | | |
| BLCA | | | | poor | | | |
| LGG | poor | poor | | good | poor | | good |
| BRCA | | good | | | | | good |
| CESA | poor | | poor | | | | |
| CCA | | | | | | | |
| COAD | | | | | | | |
| DLBC | | | good | | | | |
| GBM | | | | | | poor | |
| HNSC | poor | | poor | | poor | | good |
| KICH | | good | | | | | good |
| KIRC | poor | poor | poor | good | poor | good | good |
| KIRP | poor | poor | poor | | | | |
| LIHC | | | | | | | good |
| LUAD | poor | | poor | | | | |
| LUSC | | poor | | | | | |
| MESO | | good | good | | | | |
| OV | | | | poor | | | |
| PAAD | | poor | poor | | | | good |
| PCPG | | | | | | | |
| PRAD | | | | | | | |
| READ | | | | | poor | | |
| SARC | poor | | | | | | good |
| SKCM | | | | | | | |
| TGCT | | | | | | | |
| THCA | | poor | poor | poor | | | |
| UCS | | | | | | | |
| UCEC | | | | | | | |
| UVM | | good | good | | poor | | |

## Slide 3
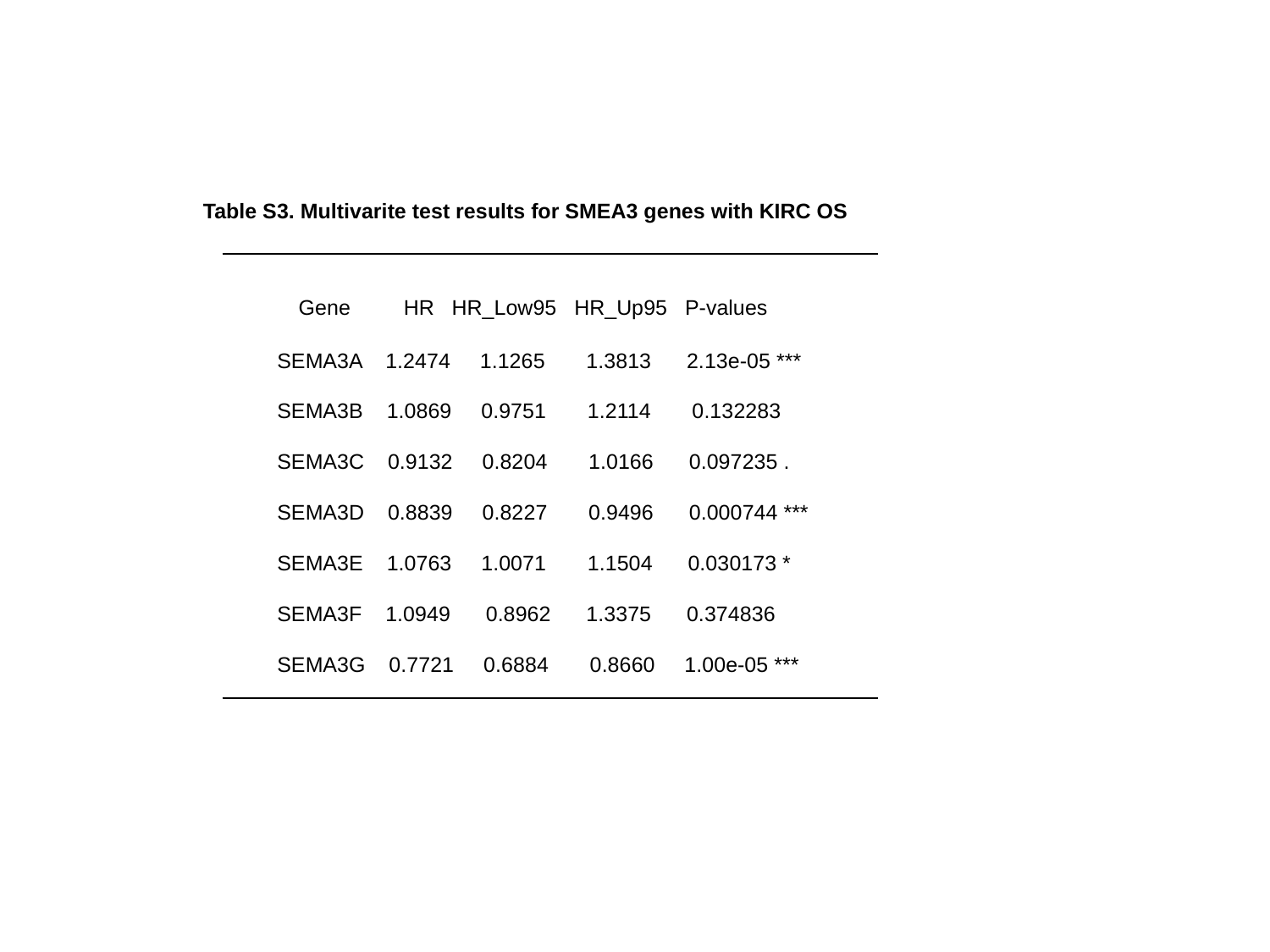

Table S3. Multivarite test results for SMEA3 genes with KIRC OS
 Gene HR HR_Low95 HR_Up95 P-values
SEMA3A 1.2474 1.1265 1.3813 2.13e-05 ***
SEMA3B 1.0869 0.9751 1.2114 0.132283
SEMA3C 0.9132 0.8204 1.0166 0.097235 .
SEMA3D 0.8839 0.8227 0.9496 0.000744 ***
SEMA3E 1.0763 1.0071 1.1504 0.030173 *
SEMA3F 1.0949 0.8962 1.3375 0.374836
SEMA3G 0.7721 0.6884 0.8660 1.00e-05 ***
