# Supplementary material for: A pan-cancer study of class-3 semaphorins as therapeutic targets in cancer
Source: BMC Med Genomics. 2020 Apr 3;13(Suppl 5):45. doi: 10.1186/s12920-020-0682-5 (PMC7118829; doi:10.1186/s12920-020-0682-5)
Supplement: Supplementary file 4 — Additional file 4: Figure S1. (A) Histogram to show the number of samples for primary tumor and adjacent normal tissues in each cancer type and the sample number for blood cancer AML. (B) Histogram to show number of death event, number of censored, and number of not available for the overall survival analysis for each cancer type. Figure S2. Expression levels of SEMA3 genes in cancerous and adjacent normal tissues for all 31 cancer types. Boxplots represent the distribution of the SEMA3 gene expression levels (log2[RSEM normalized values relative to TBP]) in primary tumour and normal tissues (if available) of different cancer types for each of the SEMA3 genes. The band inside the box is the median expression values for the gene. Comparisons between normal and tumour expression values were performed with linear mixed effects models. A p-value< 0.009 after controlling 1 false positive among all the tests was considered as significance (53). Figure S3. Kaplan-Meier survival curves to show the correlation between SEMA3 gene expression and overall survival of patients with kidney clear cell carcinoma (KIRC). Gene expression was dichotomized into “Low” and “High” based on the median expression of each specific gene in KIRC and overall survival was tested between patients with low and high gene expression using log-rank tests. Worthnoting is that in Figure S3. the p-values are different from that of the Cox-proportional hazard model where gene expression was used as a continuous variable and all 7 genes were significantly associated with overall survival of KIRC. Figure S4. (A). Kaplan-Meier survival curve to show the overall survival difference among the six immune subtypes across all cancer types. (B) and (C). Correlation matrix plots to show the association between SEMA3 gene expression and immune scores (B), and Estimate scores (C), of 22 different cancer types based on ESTIMATE algorithm. Spearman correlation was used for testing. The size of the dots stands for the a [file 12920_2020_682_MOESM4_ESM.pptx]

## Slide 1
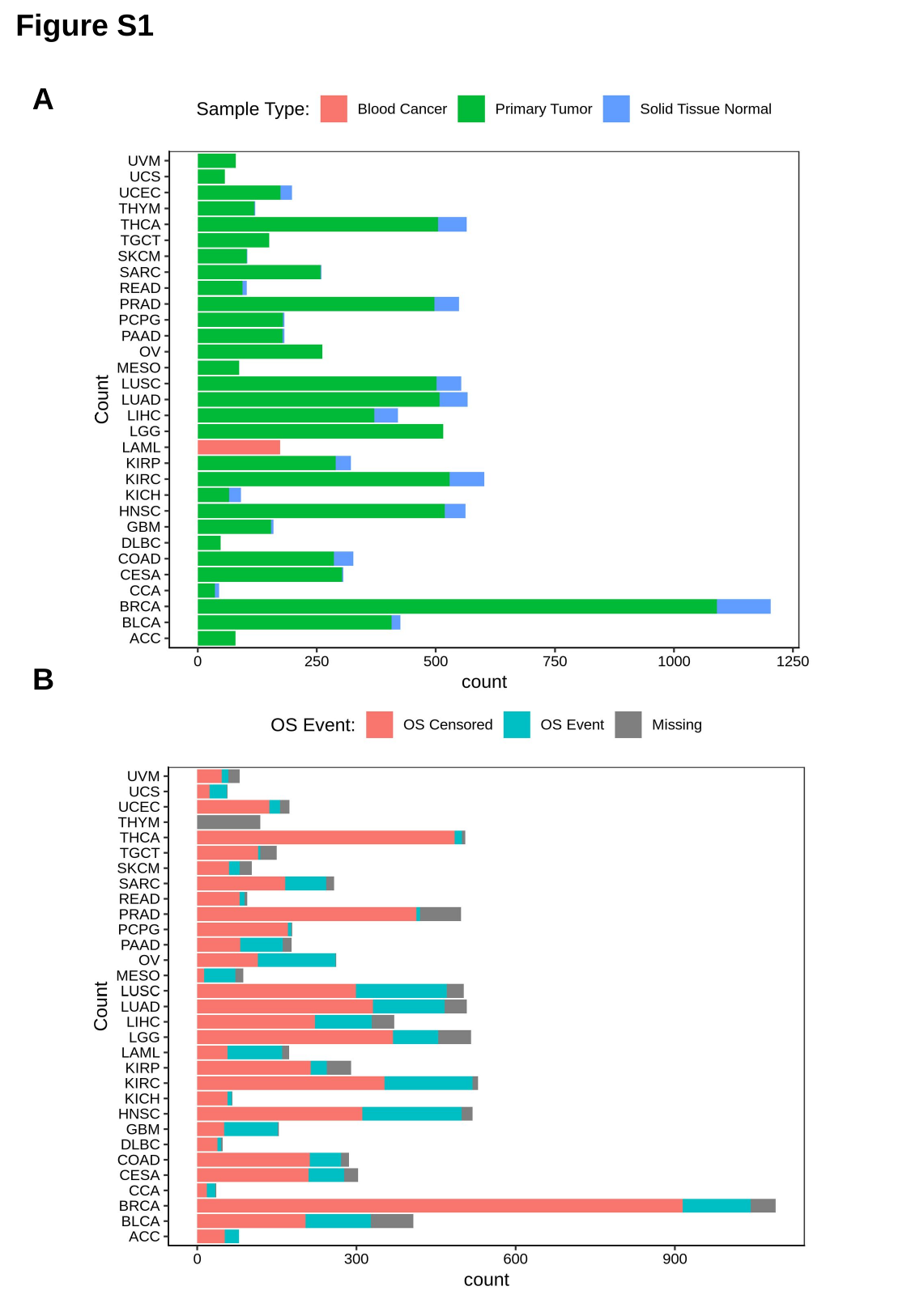

Figure S1
A
B

## Slide 2
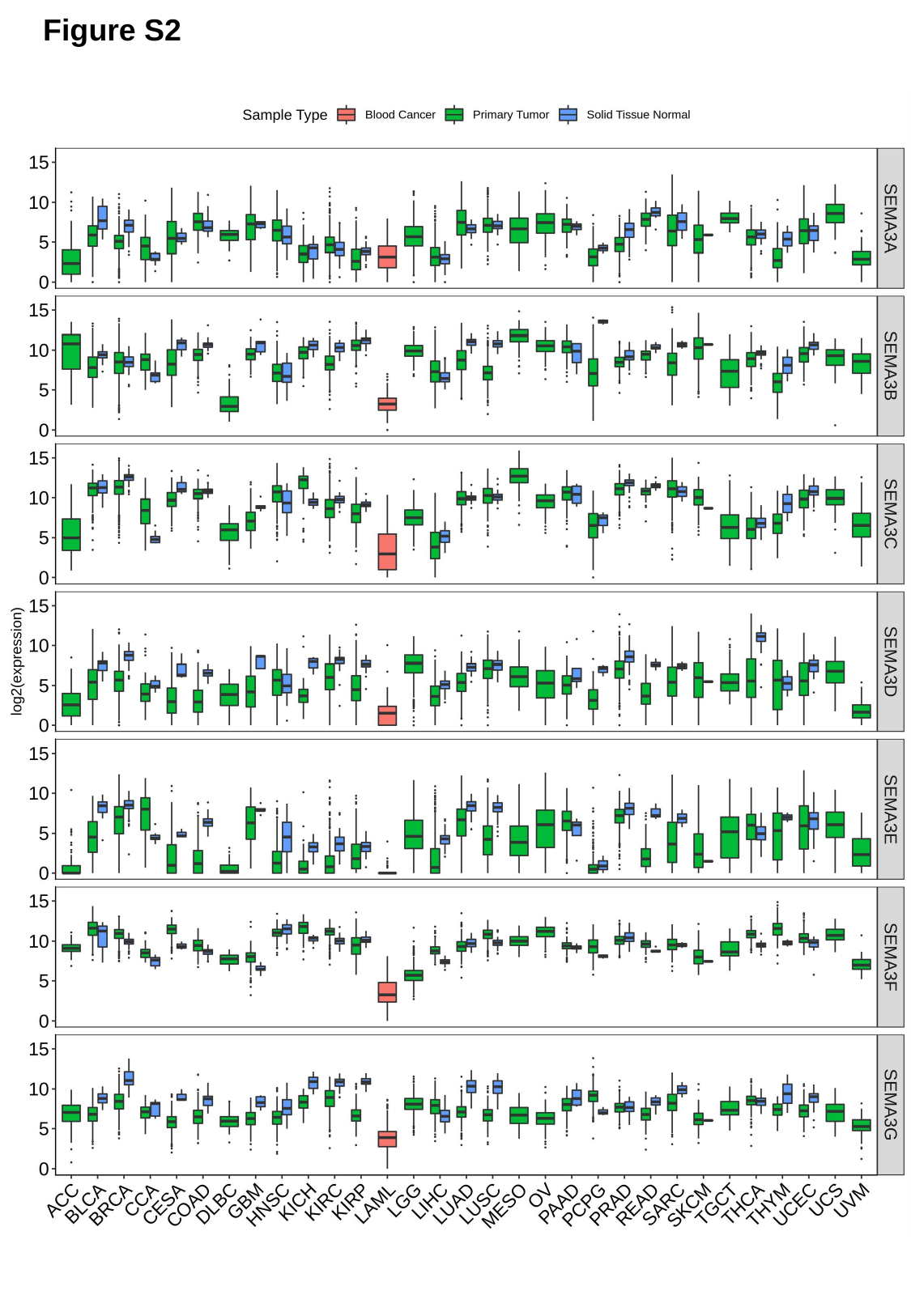

Figure S2

## Slide 3
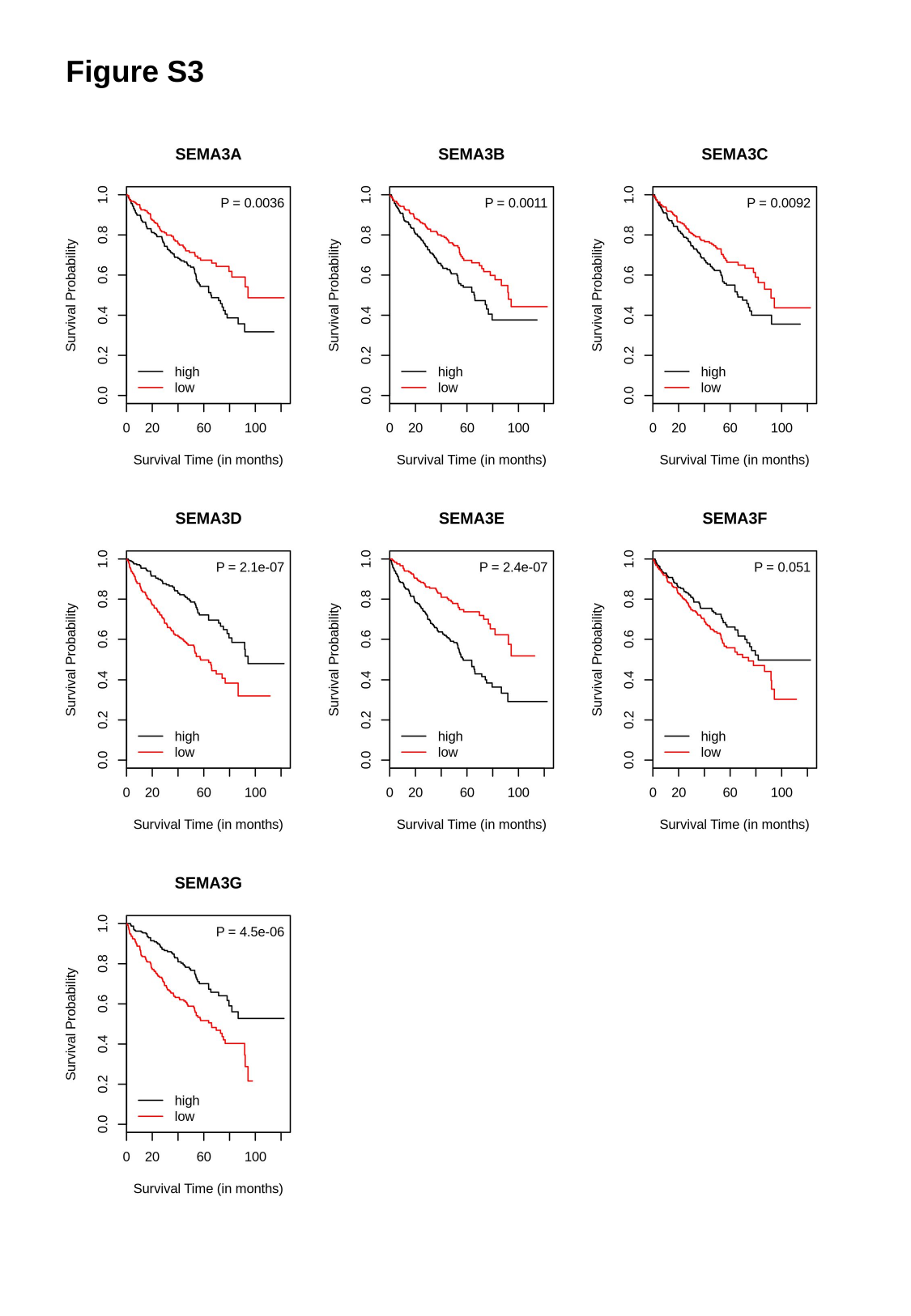

Figure S3

## Slide 4
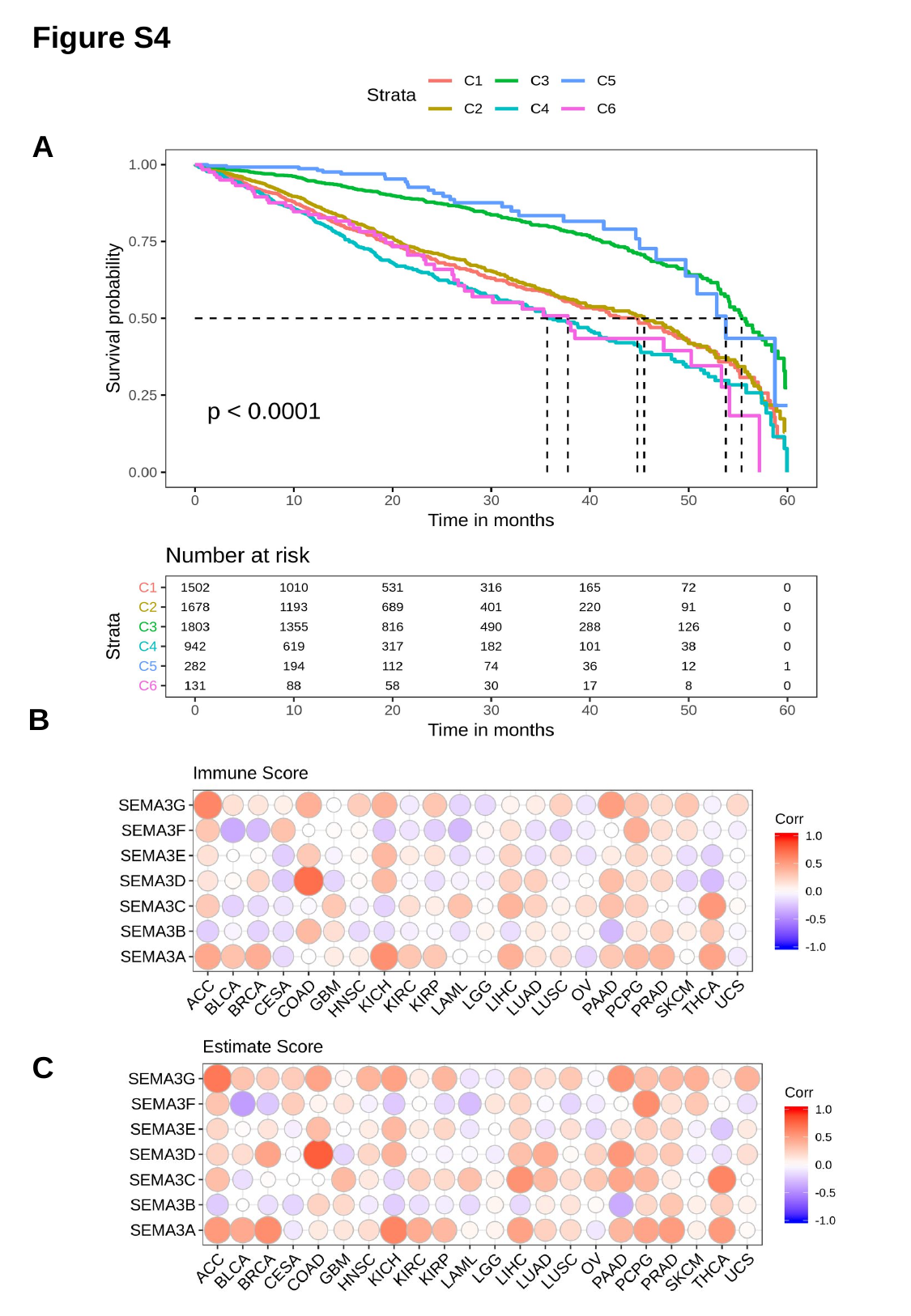

Figure S4
A
B
C

## Slide 5
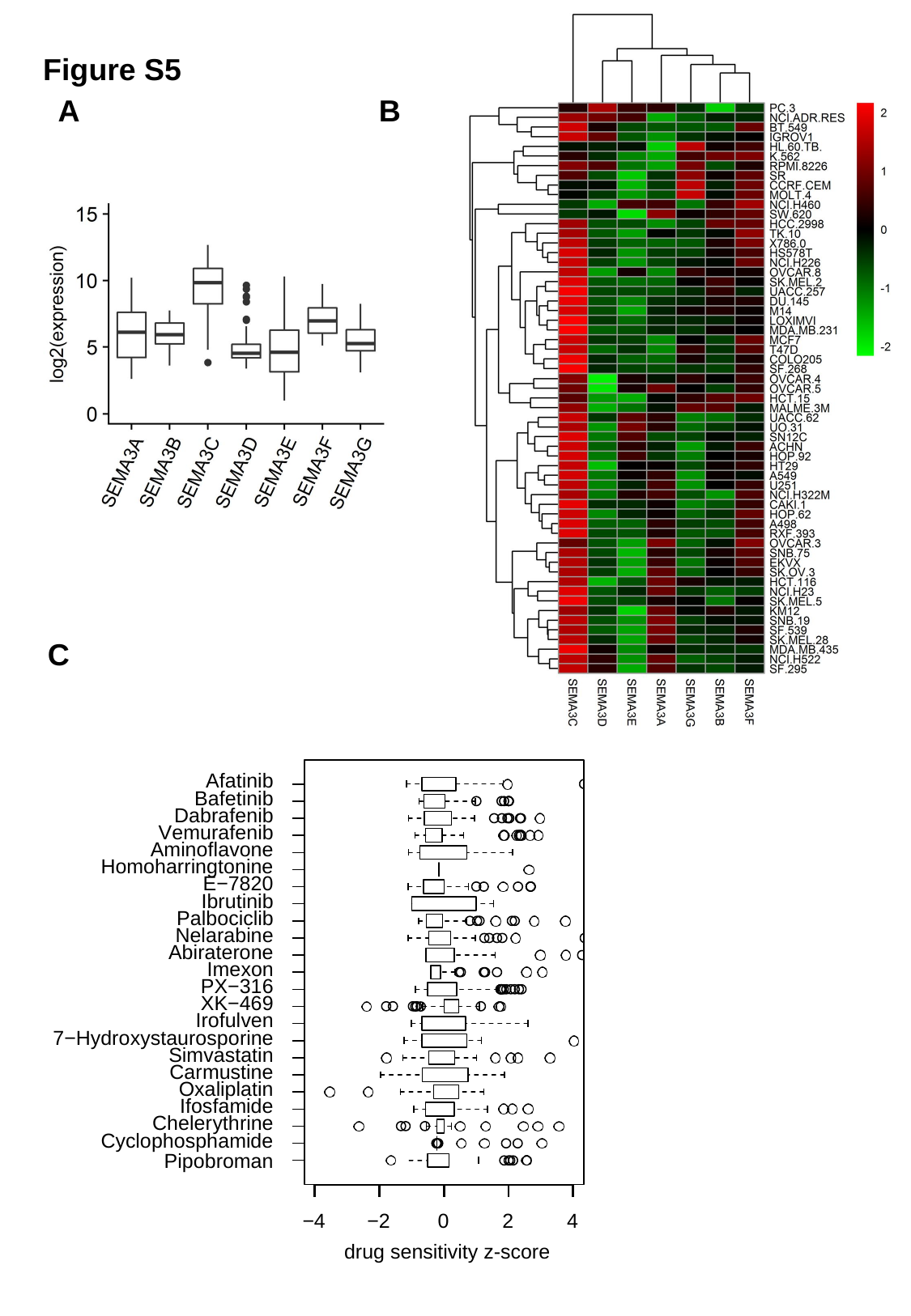

Figure S5
A
B
C
Afatinib
Bafetinib
Dabrafenib
Vemurafenib
Aminoflavone
Homoharringtonine
E−7820
Ibrutinib
Palbociclib
Nelarabine
Abiraterone
Imexon
PX−316
XK−469
Irofulven
7−Hydroxystaurosporine
Simvastatin
Carmustine
Oxaliplatin
Ifosfamide
Chelerythrine
Cyclophosphamide
Pipobroman
−4
−2
0
2
4
drug sensitivity z-score

## Slide 6
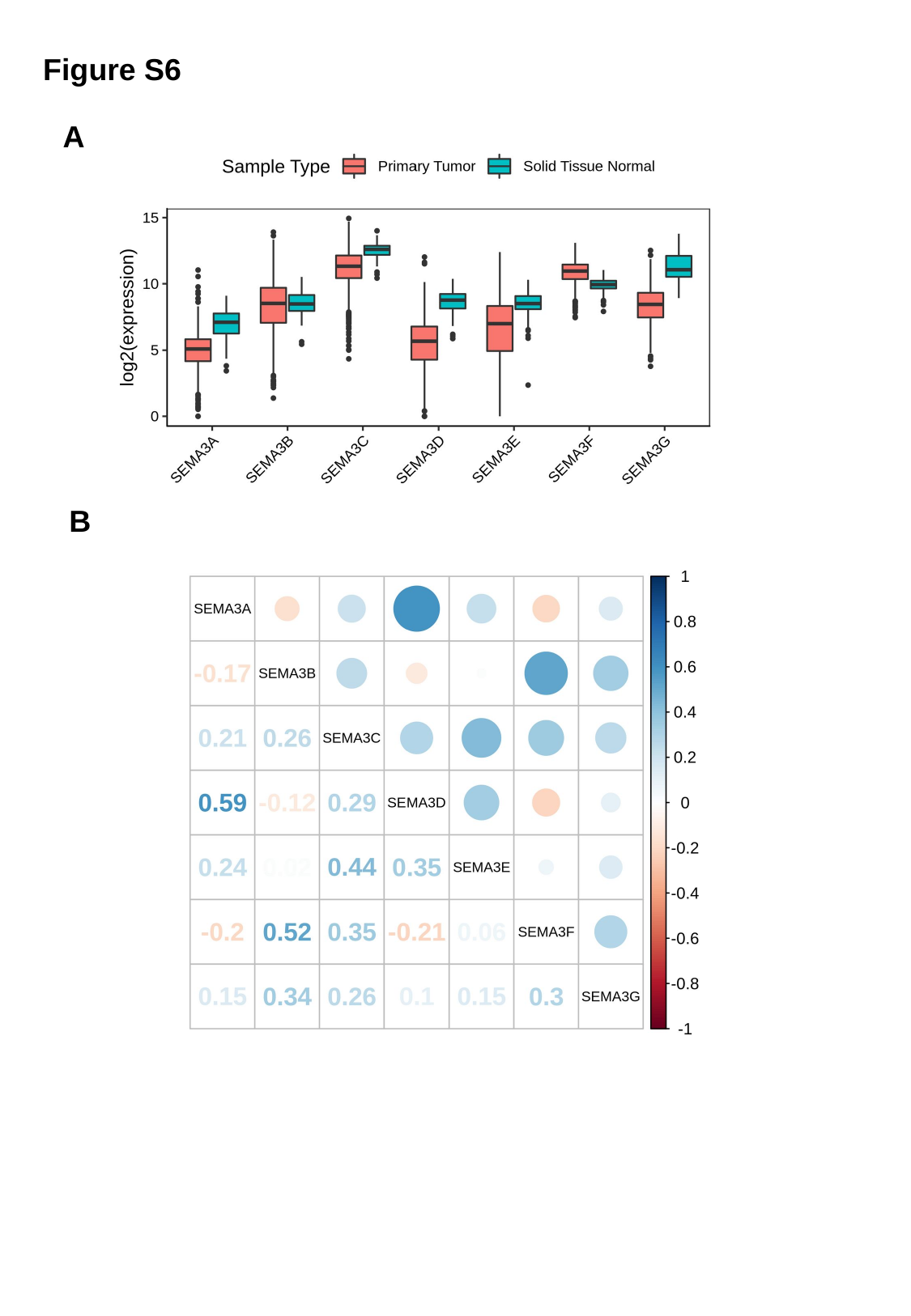

Figure S6
A
B

## Slide 7
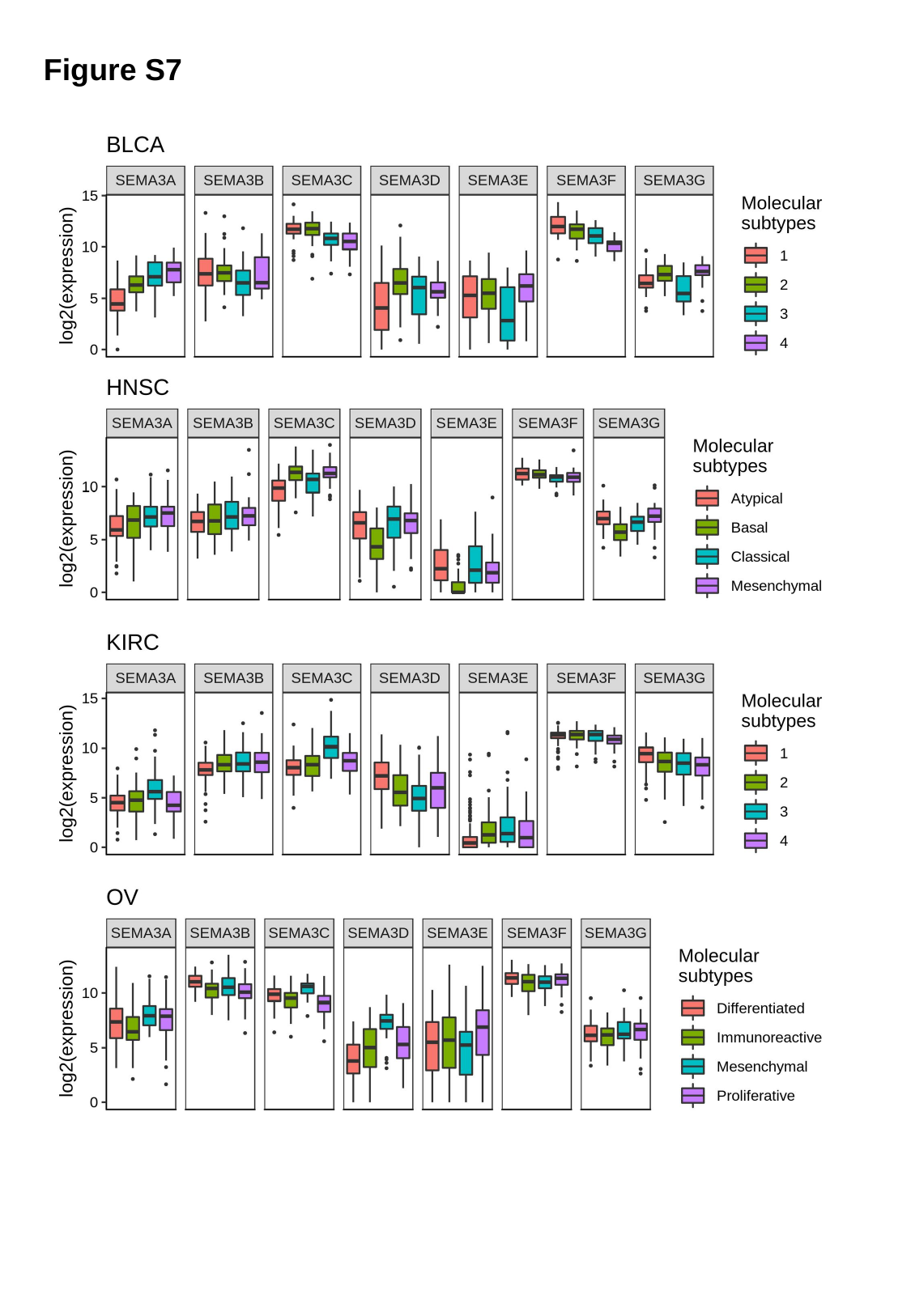

Figure S7
